# Measuring inferential importance of taxa using taxon influence indices

**DOI:** 10.1002/ece3.3941

**Published:** 2018-04-03

**Authors:** John S. S. Denton, Eric W. Goolsby

**Affiliations:** ^1^ Department of Vertebrate Paleontology American Museum of Natural History New York NY USA; ^2^ Department of Ecology and Evolutionary Biology Yale University New Haven CT USA

**Keywords:** Bayesian, taxon influence, taxon ranking, tree distance, *Tullimonstrum*

## Abstract

Assessing the importance of different taxa for inferring evolutionary history is a critical, but underutilized, aspect of systematics. Quantifying the importance of all taxa within a dataset provides an empirical measurement that can establish a ranking of extant taxa for ecological study and/or quantify the relative importance of newly announced or redescribed specimens to enable the disentangling of novelty and inferential influence. Here, we illustrate the use of taxon influence indices through analysis of both molecular and morphological datasets, introducing a modified Bayesian approach to the taxon influence index that accounts for model and topological uncertainty. Quantification of taxon influence using the Bayesian approach produced clear rankings for both dataset types. Bayesian taxon rankings differed from maximum likelihood (ML)‐derived rankings from a mitogenomic dataset, and the highest ranking taxa exhibited the largest interquartile range in influence estimate, suggesting variance in the estimate must be taken into account when the ranking of taxa is the feature of interest. Application of the Bayesian taxon influence index to a recent morphological analysis of the Tully Monster (*Tullimonstrum*) reveals that it exhibits consistently low inferential importance across two recent treatments of the taxon with alternative character codings. These results lend support to the idea that taxon influence indices may be robust to character coding and therefore effective for morphological analyses. These results underscore a need for the development of approaches to, and application of, taxon influence analyses both for the purpose of establishing robust rankings for future inquiry and for explicitly quantifying the importance of individual taxa. Quantifying the importance of individual taxa refocuses debates in morphological studies from questions of character choice/significance and taxon sampling to explicitly analytical techniques, and guides discussion of the context of new discoveries.

## 1 INTRODUCTION

A fundamental question in systematics centers on understanding the importance of different taxa for understanding phylogenetic relationships. However, quantifying taxon importance has hinged on varying definitions of the term across many biological disciplines. In conservation biology and ecology, clades have traditionally been assigned values for “phylogenetic diversity [PD]” (Faith, 1992a,b) and taxa have been assigned estimates of “originality/evolutionary distinctiveness [ED]” (Pavoine, Ollier, & Dufour, 2005; Redding et al., 2008; and sources therein), both defined using combinations of character change reconstruction or branch lengths, and node counting across clades or between taxa of interest. Computational biology has built upon these definitions of importance and has cast importance in combinatorial terms employing PD and ED as measures in a constrained optimization problem (the “Noah's Ark Problem (NAP),” a subset of the knapsack problem) to solve for the amount of unique evolutionary history that can be preserved in a subset of taxa given assumptions on an amount of funding, and the relationship of funding allocated to probability of survival (Billionnet, 2013; Hartmann & Steel, 2006; Nee & May, 1997; Weitzman, 1998).

In contrast to fixed‐tree approaches, in systematics, importance has been phrased in inferential terms, using sets of trees and either quartets or triplets (e.g., leaf and phylogenetic stability indices [Pol & Escapa, 2009; and sources therein]) or pruning to assess a taxon's effect on phylogenetic resolution (“wildcard” taxa [Nixon & Wheeler, 1992], “problematic” and “critical” taxa [Siddall, 1995], and later “rogue” and “unstable” taxa [Aberer, Krompass, & Stamatakis, 2013; Goloboff & Szumik, 2015]). Taxon importance measures emphasizing instability of taxa have been utilized predominantly to increase node support values by identifying and removing some subset of taxa from analyses, using various pipelines and optimality criteria (e.g., Aberer et al., 2013; Goloboff & Szumik, 2015).

An alternative approach, suggested by Mariadassou, Bar‐Hen, and Kishino (2012), is instead a total‐taxa approach that assigns a value called taxon influence to all taxa within a dataset based on a leave‐one‐out taxon jackknifing and reinference procedure. This approach provides a relative measure to generate ranked lists of a full set of taxa, rather than acting as a cutoff method, like rogue taxon analysis, or on subtrees, like leaf or taxon stability indices. Because a taxon influence value is derived from independent reanalysis of the nearly complete original data compared to the full original data, it is a phylogenetic inference‐based reframing of a distinctiveness measure that is derived from a full analysis rather than partitioning of a single analysis. Additionally, the generality of taxon influence methods makes them applicable to many underassessed species, for which character data, either DNA or morphology, may be the only thing known (Mace, Gittleman, & Purvis, 2003). Furthermore, unlike ED/PD measures, taxon influence analyses do not require time‐calibrated phylogenies, which frequently necessitate a degree of knowledge of the fossil and/or biogeographic record unavailable for many groups of interest. Given this broad applicability and minimal assumptions, taxon influence approaches stand to potentially bridge the gap between definitions of importance in conservation and systematics by generating minimal‐assumption taxon rankings based on whole tree inference, which may subsequently guide the acquisition of data for clades of interest that lack the kind of information necessary for NAP approaches. Furthermore, such rank lists may be useful to track changes in character data as more analyses at phylogenomic (Bragg, Potter, Bi, & Moritz, 2016; Faircloth et al., 2012) and phenomic (e.g., Copes, Lucas, Thostenson, Hoekstra, & Boyer, 2016; Goswami, 2015; O'Leary & Kaufman, 2011) scales increase in size.

Similarly, because taxon influence values are estimated for all taxa in a dataset, the relative position of a taxon of interest in the ranking of taxa may be useful for explicitly quantifying hypotheses of taxon importance implicit in many announcements of newly discovered or redescribed taxa. For example, in publications of new taxa based on phenomic data generated by tomographic methods, it remains a standard procedure to place these specimens using a parsimony analysis and to present character optimizations and contextualization of the new taxon based on its inferred position relative to other known groups on either an optimal or consensus topology (e.g., Giles, Friedman, & Brazeau, 2015; McCoy et al., 2016; Van Roy, Daley, & Briggs, 2015; Zhu et al., 2013). Such announcements are effectively verbal hypotheses of taxon importance. Despite this fact, existing inferential methods are insufficient for testing these hypotheses, because taxon importance is a relative measure that must account for both the importance of the other taxa and the effects of the characters used to infer the phylogeny.

However, two problems exist with current taxon influence implementations. First, existing implementations are based on maximum likelihood, which infers a single optimized tree topology. Influence values for a taxon derived from trees estimated using ML are therefore based on a comparison of only two topologies that are assumed to be fixed estimates. These estimates thus critically neglect uncertainty—a value as important as the tree itself (Huelsenbeck & Rannala, 2004)—an omission which stands to significantly affect the inferred influence values and rankings generated by the taxon influence procedure.

Second, existing taxon influence procedures discussed in Mariadassou et al. (2012) utilize either the Robinson‐Foulds metric (RF; Robinson and Foulds, 1981) or branch score difference (BSD; Kuhner and Felsenstein, 1994) to quantify differences between trees. Both values are derived from the computational literature and are agnostic to the issue of influential taxa. For example, the RF metric can produce maximal values for trivial rearrangements of a single taxon pair (Böcker, Canzar, & Klau, 2013; Lin, Rajan, & Moret, 2012), making it likely susceptible to the effects of rogue taxon behavior. The BSD, although accounting for both branch length and topological differences, is based on the RF metric and likely inherits this problem. Additionally, the interaction of differences in topology and branch lengths in the BSD may counteract one another in cases where short branch lengths and topological differences occur simultaneously (Kuhner & Felsenstein, 1994). A tree distance specific to questions of taxon influence remains an outstanding problem.

To address these issues and to demonstrate the utility of taxon influence analysis for both robust ranking and taxon rank placement, we apply a modified version of the original taxon influence index (TII) approach of Mariadassou et al. (2012) to three published datasets: a complete mitogenomic dataset of reptiles (Jonniaux & Kumazawa, 2008), here referred to as JK2008, and two recently published datasets debating the placement of the unusual fossil taxon *Tullimonstrum* in a phylogenetic context (McCoy et al., 2016; Sallan et al., 2017). We account for tree uncertainty using a Bayesian approach to TII calculation discussed, but not implemented, by Mariadassou et al. (2012), and also present a novel tree distance to circumvent problems with the RF metric and BSD invoked in the original publication.

## 2 METHODS

### 2.1 Phylogenetic analyses

Bayesian phylogenetic analyses were conducted in MrBayes v.3.2.6 (Ronquist et al., 2012). The JK2008 dataset was analyzed using the same model parameterization (GTR + I + Γ) as in Mariadassou et al. (2012). Analysis was run using a single chain of 10 million generations, with a 20% burn‐in. The *Tullimonstrum* datasets were analyzed using the Mkv + Γ model (Lewis, 2001) with six discrete classes, using a single chain of 20 million generations, with a 50% burn‐in. In both cases, the number of generations required to reach a sufficient topological ESS was determined by calculation of approximate ESS values in the R package *rwty* (Warren, Geneva, & Lanfear, 2017). Because the TII approach is a single taxon‐pruning procedure, all jackknifed analyses were assigned the parent number of generations.

### 2.2 Taxon influence measurement

The taxon influence index (TII), the expected distance between pairs of trees in the posterior distribution, was calculated according to Mariadassou et al. (2012):

$$\text{TII}\left(i\right)=E\left[d\left(T_{i}^{*},T_{i}^{\prime }\right)\right]=\sum_{T^{*},T^{\prime }}w_{i}\left(T^{*}\right)w_{i}\left(T^{\prime }\right)d\left(T^{*},T^{\prime }\right)$$

where T* is the posterior distribution of trees from analysis using all taxa, *T*′ is a posterior distribution of trees in which a focal taxon is dropped before analysis, *T* ^′^_i_ is a phylogenetic tree from a posterior *T*′ for which taxon *i* was dropped before analysis, *T* ^*^_i_ is a tree from the posterior T* in which taxon *i* was dropped *a posteriori* for comparison with *T* ^′^_i_, *w*_*i*_ is the posterior probability of a tree *i*, and *d*(●,●) is a topological distance between the two trees. The original calculation from Mariadassou et al. (2012) was modified in two ways.

First, because comparisons between posterior distributions of trees based on pairwise distances between elements necessitate $\left(\begin{array}{c} n\\ 2 \end{array}\right)$ summations, where *n* is the number of unique postburn‐in topologies, to fully compare the high‐dimensional posterior, variance in the TII value due to a finite approximation with a smaller number of sums was estimated by resampling. For each iteration, a number of trees equal to min(|T*|,|*T*′|) were sampled without replacement from each posterior according to their posterior probabilities (*w*_*i*_), and this sample was used to calculate the TII for each of 100 iterations. The estimated TII value for each taxon was the median of these resampled values.

Second, given the potential issues with both the RF metric and BSD regarding influential taxa, informative distances between trees were defined as the ratio of the distance between the trees to the size of the shared tree. This new criterion was satisfied by a value referred to here as the SPR excess, an SPR distance—the minimum number of subtree‐pruning and regrafting rearrangements required to turn one tree into another (e.g., Goloboff, 2008)—scaled by the number of taxa in the maximum agreement subtree (MAST, (Gordon, 1979; Finden & Gordon, 1985; Valiente, 2009), and see Ge, Wang, and Kim, 2005 for an example of the implications of deviation in tree shapes between a difference and similarity measure in the context of molecular data).

Finally, for comparison to TII estimates, a rogue taxon analysis (Aberer, Pattengale, & Stamatakis, 2010; Aberer et al., 2013) using the Mkv + Γ model was conducted in raxml v8.2.9 (Stamatakis, 2014). To standardize the comparison to a fixed set of trees, the postburn‐in distribution of trees from the Bayesian analysis, rather than a collection of bootstrap trees, was used. All TII calculations were conducted using scripts written by the authors ([Link], [Link], [Link], [Link], [Link], [Link], [Link], [Link], [Link]) in the R environment (R Core Team 2016) using the *ape* (Paradis, Claude, & Strimmer, 2004), *phangorn* (Schliep, 2011), *stringr* (Wickham, 2015), and *gespeR* (Schmich et al., 2015) packages. Differences in taxon influence‐based rankings between the two *Tullimonstrum* datasets, and differences in rank by proportion of missing data, were calculated for this dataset using rank‐biased overlap (Webber, Moffat, & Zobel, 2010), for which significance was assessed using a permutation procedure against the null hypothesis of dissimilar rankings.

## 3 RESULTS

### 3.1 Phylogenetic trees

**Figure 1:**
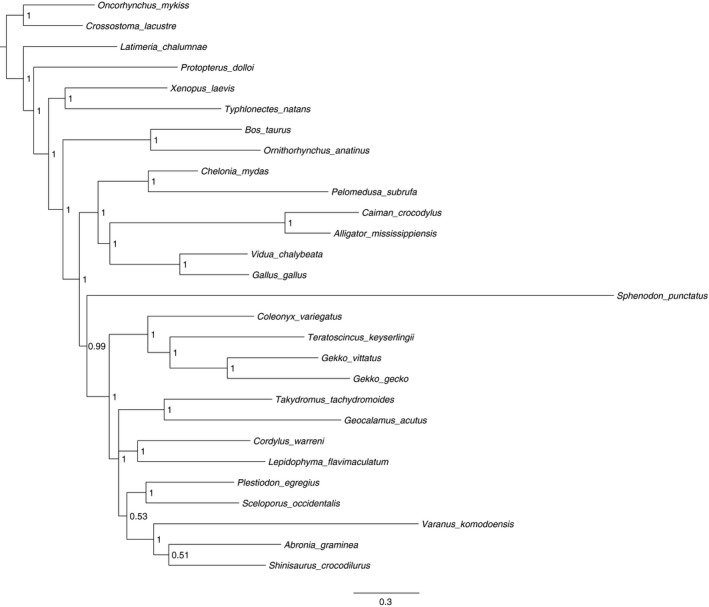
Results of Bayesian analysis of the JK2008 dataset (50% majority‐rule consensus tree), with clade credibility values displayed at nodes. The topology was the same as that recovered by Mariadassou et al. (2012) after accounting for clade collapse procedures

Phylogenetic analysis of the Jonniaux and Kumaza (JK2008) dataset demonstrated convergence in the postburn‐in tree topology (approxESS_T_ > 500) and ESS > 200 for all model parameters. The 50% majority‐rule consensus tree (Figure 1) revealed high clade support values throughout most of the tree, with low support values in the same locations as those inferred for bootstrap values by Mariadassou et al. (2012) for this dataset. The 50% majority‐rule consensus topology inferred under the Bayesian analysis was identical to that inferred by Mariadassou et al. (2012) under maximum likelihood, with the exception of the procedural collapse of the consensus tree for regions where clade support values were under 50%. There were 36 unique trees in the postburn‐in posterior distribution. The 99% credibility interval contained ten of these trees.

**Figure 2:**
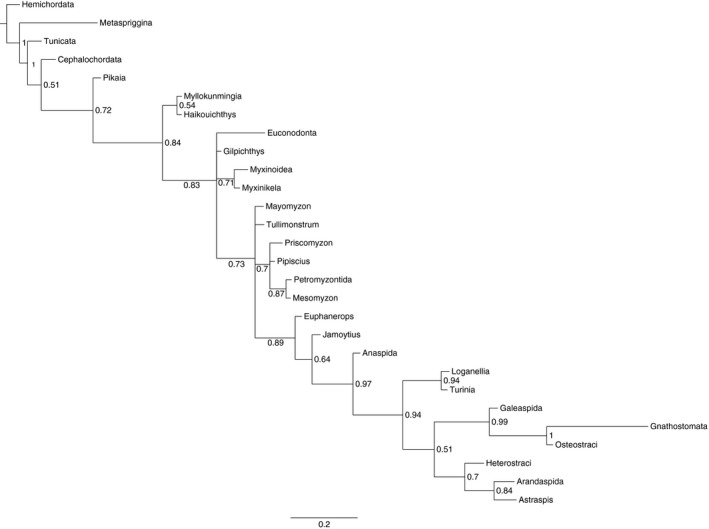
Results of Bayesian analysis of the McCoy et al. *Tullimonstrum* dataset (50% majority‐rule consensus tree), with clade credibility values displayed at nodes. Differences in topology between this analysis and the results of McCoy et al. are described in the text

Phylogenetic analysis of the *Tullimonstrum* dataset demonstrated convergence in the postburn‐in tree topology (approxESS_T_ ~ 291), and ESS > 200 for all model parameters. The postburn‐in posterior distribution contained 9877 unique trees. The 50% majority‐rule consensus tree (Figure 2) differed significantly from the parsimony analysis of McCoy et al. (2016) in several ways. First, *Metaspriggina* was recovered as sister to the remaining ingroup taxa, with high support (1.0). Second, *Tunicata* was recovered as diverging before *Cephalochordata*, with high support (1.0). Third, the locations of the polytomies within the tree were shifted. A clade of *Haikuichthys *+ *Myllokunmingia* was recovered as sister to the remaining taxa, with low support (0.54). In the remaining taxa, *Euconodonta, Gilpichthys,* a clade of *Myxinoidea* + *Myxinikela* (support 0.71), and the remaining taxa were recovered in a central polytomy. Within the remaining taxa, *Mayomyzon*,* Tullimonstrum*, a clade of *Priscomyzon, Pipiscius, Petromyzontida,* and *Mesomyzon* (support 0.7) were recovered in a polytomy with the remaining taxa. The remaining taxa exhibited the same phylogenetic structure as in McCoy et al. (2016).

### 3.2 Taxon influence values

**Figure 3:**
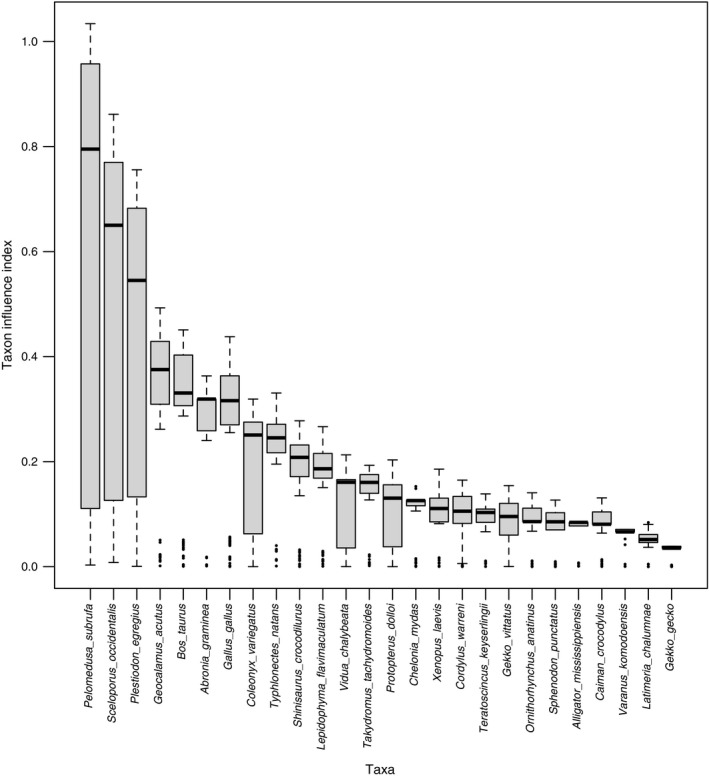
Results of taxon influence analysis of the JK2008 dataset. Rankings exhibited well‐separated medians and non‐normally distributed estimate distributions, with an apparent relationship between interquartile range and median value and downward‐directed outliers

TII analysis of the JK2008 dataset produced mostly well‐separated median values, with a small number of downwardly directed outliers, an apparent negative relationship between taxon influence and the interquartile range of the TII estimate, and no apparent relationship between TII estimate and the skewness of the distribution (Figure 3).

The medians of the three highest ranking taxa (*Pelomedusa subrufa*,* Sceloporus occidentalis*, and *Plestiodon egregius*) were well separated both from each other and from the other taxa. These taxa differed from those ranked highest by Mariadassou et al. (2012) under maximum likelihood using the BSD (*Shinisaurus crocodilus*,* Coleonyx variegatus*, and *Sceloporus occidentalis*). Four of the eight ingroup taxa identified as influential by Mariadassou et al. (2012) were recovered in the top of the Bayesian ranking (*Geocalamus acutus, Sceloporus occidentalis, Coleonyx variegatus,* and *Pelomedusa subrufa*).

**Figure 4:**
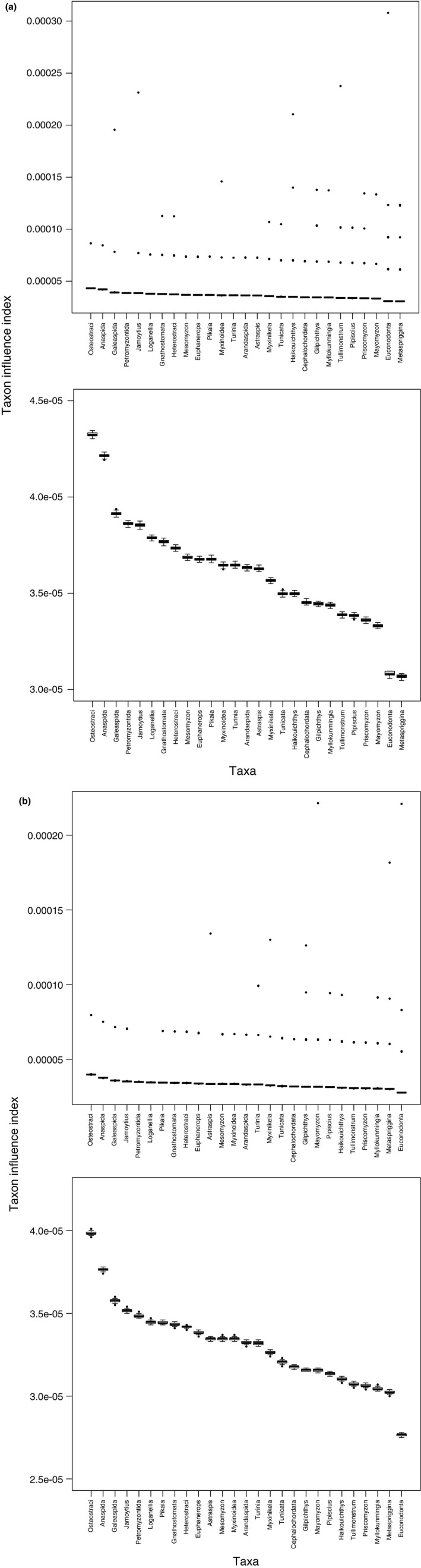
(a) Results of taxon influence analysis of the McCoy et al. dataset. Upper plot shows full range of estimates, including upwardly directed outliers, which comprised no more than 10% of TII estimates per taxon. Lower plot shows TII rankings excluding outliers, with well‐separated median values. *Tullimonstrum* falls out in the lower quartile of taxon influence for all ingroup taxa. (b) Results of taxon influence analysis of the Sallan et al. dataset, with different interpretations of eight *Tullimonstrum* character states. Upper plot and lower plot as in Figure 4a. *Tullimonstrum* falls out in the lower quartile of influence values

**Figure 5:**
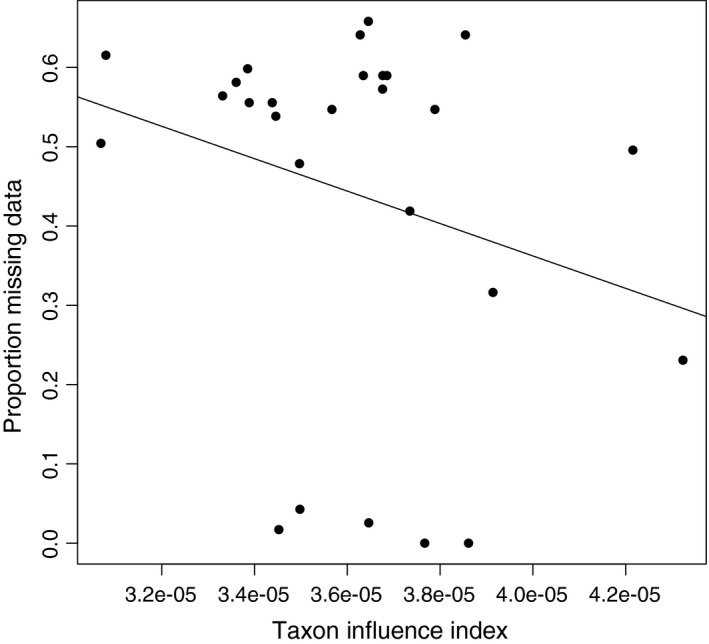
Scatterplot of inferred taxon influence values versus the proportion of missing data in the taxon for the McCoy et al. dataset. There was no significant relationship between influence value and proportion of missing data (*R* ^2^ = .02; *p* = .19). Ranking based on the proportion of missing data was unrelated to the TII‐based ranking (rbo = 0.189; *p* = .829)

TII analysis of the *Tullimonstrum* datasets produced well‐separated values (Figure 4a,b, lower), with a small number of extreme and directionally biased outliers that comprised no more than 10% of each taxon's TII estimates (Figure 4a,b, upper). Both analyses placed *Tullimonstrum* in the lower quartile of taxon influence (median TII_McCoy_ = 3.38e−05; median TII_Sallan_ = 3.07e−05) for all 27 ingroup taxa. Estimated TII values were lower in the Sallan et al. (2017) dataset than in the McCoy et al. (2016) dataset, and rankings exhibited several differences in the middle and tail of the list. However, the null hypothesis of dissimilarity in the rankings was rejected (rbo = 0.932, *p* < .0001). Rogue taxon analysis did not identify any taxa to be pruned. The ranking of taxa based on proportion of missing values (“?”; Figure 5) was unrelated to the estimated TII‐based ranks (rbo = 0.189; *p* = .829).

## 4 DISCUSSION

Inference of well‐separated TII values for two contrasting data types—molecular data and morphological data—and for differing degrees of phylogenetic signal suggests the Bayesian‐based approach presented here is robust and applicable for ranking taxa with different data properties. Additionally, the stability in rank location of a focal taxon (*Tullimonstrum*) using our approach suggests the method may be beneficial for contextualizing hypotheses of the importance of individual taxa using analytical rank results.

### 4.1 Molecular dataset

The difference in taxon ranks between the present analysis and the original ML analysis underscores the important distinction between the two methods. Although the two approaches exhibited some overlap in highly ranked taxa (Figure 3; Mariadassou et al., 2012; Figures 4 and 5), the interquartile ranges around the median TII estimates in the present analysis reveal that TII‐based taxon rankings are likely to be significantly influenced by topological uncertainty. This implication is supported by the apparent disconnect between the strongly peaked posterior distribution, suggesting strong phylogenetic signal, and the variably wide and skewed shapes of the TII distributions for each taxon in the JK2008 dataset. The importance of the shape of TII distributions is further underscored by the overlap in interquartile range of the three highest ranking taxa with the medians of those taxa identified as highest rank by Mariadassou et al. (2012).

Alternatively, such distributions may reflect an analytical artifact, such as model choice. Mariadassou et al. (2012) observed some differences among TII estimates and rankings when different models were employed on an amino acid dataset. Although there was an apparent relationship between TII median and interquartile range (Figure 3), it is currently unclear whether this variation is itself a feature of taxa that may reflect some degree of rogue behavior, or whether it is a computational artifact that may change with the number of TII sampling replicates, or with MCMC search intensity or model choice. We presented 100 iterations as a starting value for resampling the TII estimates, but more may be necessary for certain datasets. However, given the potential of Bayesian methods for obviating model selection through procedures like reversible‐jump MCMC (Ronquist et al., 2012), which is not applicable in commonly used maximum likelihood phylogenetic inference programs, model choice may not affect Bayesian TII estimates and rankings as strongly.

### 4.2 Morphological dataset

The ranking of *Tullimonstrum* using methods like taxon influence is significant because it reframes the debate in the recent literature on the taxon (Clements et al., 2016; McCoy et al., 2016; Sallan et al., 2017) from a conceptual one of character choice/significance and taxon sampling to an explicitly analytical one of the inferential importance of the taxon relative to other taxa. Specifically, based on the present results (Figures 4 and 5), we conclude that, relative to the selected taxa and characters, *Tullimonstrum* does not have a significant effect on our inference of the shape of evolutionary history; it is not inferentially important relative to the dataset. The hypothesis that *Tullimonstrum* is important, implied in the original paper (McCoy et al., 2016), is by this measure rejected, a conclusion that is further supported by the robustness of the *Tullimonstrum* rank position to differences in the coding of eight disputed character states between the McCoy et al. and Sallan et al. datasets.

This conclusion stands in contrast to the intuitive idea of importance as suggested by the many apparently unique features in the taxon, notably the proboscis and eyestalks, and its placement within lampreys in parsimony analysis (McCoy et al., 2016). This unexpected outcome reveals that a distinction must be made between novelty and inference when contextualizing new taxonomic discoveries or redescriptions. Taxon influence analysis makes this distinction possible by explicitly quantifying one element (inferential importance).

Additionally, given that the taxon influence measure accounts for taxon and character sampling, the low rank inferred for *Tullimonstrum* may be an artifact of sampling design incurred by adding new taxa to existing character matrices (see, for example, [Davis, Finarelli, & Coates, 2012; Zhu et al., 2013; Giles et al., 2015] and [McCoy et al., 2016; Morris & Caron, 2014; Sansom, Freedman, Gabbott, Aldridge, & Purnell, 2010]). Future work may utilize taxon influence measures to address the idea of refinability in morphological character datasets.

### 4.3 Methodological implications and future directions

The bounds around the resampling results (Figures 3 and 4) suggest that the finite sum approximation utilized in this study generates reproducible rankings of taxon influence and may thus be an effective approximation for calculating taxon influence based on the SPR excess distance measure, from posterior distributions of trees for which the probabilities were calculated using the standard approach. The causes for the existence of directional outliers in the studied datasets (Figures 3 and 4) is currently unclear, but may be an artifact of either the number of finite sums, or of an interaction between the probabilities of trees and the SPR distances between them.

Although we have focused on several standard parametric models for nucleotide substitution and morphological character transformation, other posterior distributions of trees are possible. It may be useful to, for example, explore the distribution of parsimony‐score‐ranked trees under the Bayesian approach using the TS97/no common mechanism model (Tuffley & Steel, 1997), for comparison with the results of parametric models, or as a heuristic for larger datasets. It may also prove worthwhile to calculate tree posteriors using information‐theoretic measures (Larget, 2013; Lewis et al., 2016) and nonuniform tree priors, which may reveal a more universal metric for taxon influence assessment. Finally, although our method assesses the influence of individual taxa using a leave‐one‐out jackknifing approach as an intuitive method for generating ranked lists of taxa based on what is essentially the “main effect” of each taxon, the contributions of higher‐order “interaction” effects, such as pairwise‐ or clade‐based influence, have yet to be addressed by the taxon influence approach. Approaches for estimating clade stability have been discussed by several authors, including Pol and Escapa (2009), for reduced positional congruence, and Gatesy (2000), for linked branch support. In these cases, analyses were conducted on complete‐taxon datasets and sets of most parsimonious trees, rather than via a taxon jackknifing approach. The theory for, and effect of, pairwise or higher‐order interactions on taxon influence values is currently unclear. Future work expanding the taxon influence method through a leave‐*k*‐out approach may be beneficial, although direct interpretation of the results of complex multi‐taxon interaction may be difficult.

## CONFLICT OF INTERESTS

We declare we have no competing interests.

## AUTHOR CONTRIBUTIONS

J.S.S.D. conceived the study, wrote the original taxon influence code, and drafted the manuscript. E.W.G. wrote the tree parser, refined the code, ran cluster‐based analyses, and edited the manuscript. Both authors approved submission.

## DATA ACCESSIBILITY

All scripts and functions for calculating taxon influence and associated analyses are provided as [Link], [Link], [Link], [Link], [Link], [Link], [Link], [Link], [Link] accompanying this paper.

## ETHICAL STATEMENT

The research complies with all national and international ethical requirements.

## References

[ece33941-bib-0001] Aberer, A. J. , Krompass, D. , & Stamatakis, A. (2013). Pruning rogue taxa improves phylogenetic accuracy: An efficient algorithm and webservice. Systematic Biology, 62, 162–166. https://doi.org/10.1093/sysbio/sys078 22962004 10.1093/sysbio/sys078 PMC3526802

[ece33941-bib-0002] Aberer, A. J. , Pattengale, N. D. , & Stamatakis, A. (2010). Parallelized phylogenetic post‐analysis on multi‐core architectures. Journal of Computational Science, 1, 107–114. https://doi.org/10.1016/j.jocs.2010.03.006

[ece33941-bib-0003] Billionnet, A. (2013). Solution of the generalized Noah's Ark problem. Systematic Biology, 62, 147–156. https://doi.org/10.1093/sysbio/sys081 22997022 10.1093/sysbio/sys081

[ece33941-bib-0004] Böcker, S. , Canzar, S. , & Klau, G. W. (2013). The generalized Robinson‐Foulds metric In International Workshop on Algorithms in Bioinformatics (pp. 156–169). New York, NY: Springer.

[ece33941-bib-0005] Bragg, J. G. , Potter, S. , Bi, K. , & Moritz, C. (2016). Exon capture phylogenomics: Efficacy across scales of divergence. Molecular Ecology Resources, 16, 1059–1068. https://doi.org/10.1111/1755-0998.12449 26215687 10.1111/1755-0998.12449

[ece33941-bib-0006] Clements, T. , Dolocan, A. , Martin, P. , Purnell, M. A. , Vinther, J. , & Gabbott, S. E. (2016). The eyes of *Tullimonstrum* reveal a vertebrate affinity. Nature, 532, 500–503. https://doi.org/10.1038/nature17647 27074512 10.1038/nature17647

[ece33941-bib-0007] Copes, L. E. , Lucas, L. M. , Thostenson, J. O. , Hoekstra, H. E. , & Boyer, D. M. (2016). A collection of non‐human primate computed tomography scans housed in MorphoSource, a repository for 3D data. Scientific Data, 3, 160001 https://doi.org/10.1038/sdata.2016.1 26836025 10.1038/sdata.2016.1 PMC4736502

[ece33941-bib-0008] Davis, S. P. , Finarelli, J. A. , & Coates, M. I. (2012). Acanthodes and shark‐like conditions in the last common ancestor of modern gnathostomes. Nature, 486, 247–250. https://doi.org/10.1038/nature11080 22699617 10.1038/nature11080

[ece33941-bib-0009] Faircloth, B. C. , McCormack, J. E. , Crawford, N. G. , Harvey, M. G. , Brumfield, R. T. , & Glenn, T. C. (2012). Ultraconserved elements anchor thousands of genetic markers spanning multiple evolutionary timescales. Systematic Biology, 61, 717–726. https://doi.org/10.1093/sysbio/sys004 22232343 10.1093/sysbio/sys004

[ece33941-bib-0010] Faith, D. P. (1992a). Conservation evaluation and phylogenetic diversity. Biological Conservation, 61, 1–10. https://doi.org/10.1016/0006-3207(92)91201-3

[ece33941-bib-0011] Faith, D. P. (1992b). Systematics and conservation: On predicting the feature diversity of subsets of taxa. Cladistics, 8, 361–373. https://doi.org/10.1111/j.1096-0031.1992.tb00078.x 10.1111/j.1096-0031.1992.tb00078.x 34929967

[ece33941-bib-0012] Finden, C. , & Gordon, A. (1985). Obtaining common pruned trees. Journal of Classification, 2, 255–276. https://doi.org/10.1007/BF01908078

[ece33941-bib-0013] Gatesy, J. (2000). Linked branch support and tree stability. Systematic Biology, 49, 800–807. https://doi.org/10.1080/106351500750049842 12116441 10.1080/106351500750049842

[ece33941-bib-0014] Ge, F. , Wang, L.‐S. , & Kim, J. (2005). The cobweb of life revealed by genome‐scale estimates of horizontal gene transfer. PLoS Biology, 3, e316 https://doi.org/10.1371/journal.pbio.0030316 16122348 10.1371/journal.pbio.0030316 PMC1233574

[ece33941-bib-0015] Giles, S. , Friedman, M. , & Brazeau, M. D. (2015). Osteichthyan‐like cranial conditions in an Early Devonian stem gnathostome. Nature, 520, 82–85. https://doi.org/10.1038/nature14065 25581798 10.1038/nature14065 PMC5536226

[ece33941-bib-0016] Goloboff, P. A. (2008). Calculating SPR distances between trees. Cladistics, 24, 591–597. https://doi.org/10.1111/j.1096-0031.2007.00189.x 10.1111/j.1096-0031.2007.00189.x 34879631

[ece33941-bib-0017] Goloboff, P. A. , & Szumik, C. A. (2015). Identifying unstable taxa: Efficient implementation of triplet‐based measures of stability, and comparison with Phyutility and RogueNaRok. Molecular Phylogenetics and Evolution, 88, 93–104. https://doi.org/10.1016/j.ympev.2015.04.003 25865266 10.1016/j.ympev.2015.04.003

[ece33941-bib-0018] Gordon, A. (1979). A measure of the agreement between rankings. Biometrika, 66, 7–15. https://doi.org/10.1093/biomet/66.1.7

[ece33941-bib-0019] Goswami, A. (2015). Phenome10K: a free online repository for 3‐D scans of biological and palaeontological specimens. Retrieved from http://www.phenome10k.org.

[ece33941-bib-0020] Hartmann, K. , & Steel, M. (2006). Maximizing phylogenetic diversity in biodiversity conservation: Greedy solutions to the Noah's Ark problem. Systematic Biology, 55, 644–651. https://doi.org/10.1080/10635150600873876 16969940 10.1080/10635150600873876

[ece33941-bib-0021] Huelsenbeck, J. P. , & Rannala, B. (2004). Frequentist properties of Bayesian posterior probabilities of phylogenetic trees under simple and complex substitution models. Systematic Biology, 53, 904–913. https://doi.org/10.1080/10635150490522629 15764559 10.1080/10635150490522629

[ece33941-bib-0022] Jonniaux, P. , & Kumazawa, Y. (2008). Molecular phylogenetic and dating analyses using mitochondrial DNA sequences of eyelid geckos (Squamata: Eublepharidae). Gene, 407, 105–115. https://doi.org/10.1016/j.gene.2007.09.023 18029117 10.1016/j.gene.2007.09.023

[ece33941-bib-0023] Kuhner, M. K. , & Felsenstein, J. (1994). A simulation comparison of phylogeny algorithms under equal and unequal evolutionary rates. Molecular Biology and Evolution, 11, 459–468. https://doi.org/10.1093/oxfordjournals.molbev.a040126 8015439 10.1093/oxfordjournals.molbev.a040126

[ece33941-bib-0024] Larget, B. (2013). The estimation of tree posterior probabilities using conditional clade probability distributions. Systematic Biology, 62, 501–511. https://doi.org/10.1093/sysbio/syt014 23479066 10.1093/sysbio/syt014 PMC3676676

[ece33941-bib-0025] Lewis, P. O. (2001). A likelihood approach to estimating phylogeny from discrete morphological character data. Systematic Biology, 50, 913–925. https://doi.org/10.1080/106351501753462876 12116640 10.1080/106351501753462876

[ece33941-bib-0026] Lewis, P. O. , Chen, M.‐H. , Kuo, L. , Lewis, L. A. , Fučíková, K. , Neupane, S. , … Shi, D. (2016). Estimating Bayesian phylogenetic information content. Systematic Biology, 65, 1009–1023. https://doi.org/10.1093/sysbio/syw042 27155008 10.1093/sysbio/syw042 PMC5066063

[ece33941-bib-0027] Lin, Y. , Rajan, V. , & Moret, B. M. (2012). A metric for phylogenetic trees based on matching. IEEE/ACM Transactions on Computational Biology and Bioinformatics, 9, 1014–1022. https://doi.org/10.1109/TCBB.2011.157 22184263 10.1109/TCBB.2011.157

[ece33941-bib-0028] Mace, G. M. , Gittleman, J. L. , & Purvis, A. (2003). Preserving the tree of life. Science, 300, 1707–1709. https://doi.org/10.1126/science.1085510 12805539 10.1126/science.1085510

[ece33941-bib-0029] Mariadassou, M. , Bar‐Hen, A. , & Kishino, H. (2012). Taxon influence index: Assessing taxon‐induced incongruities in phylogenetic inference. Systematic Biology, 61, 337–345. https://doi.org/10.1093/sysbio/syr129 22228800 10.1093/sysbio/syr129

[ece33941-bib-0030] McCoy, V. E. , Saupe, E. E. , Lamsdell, J. C. , Tarhan, L. G. , McMahon, S. , Lidgard, S. , … Finney, L. (2016). The ‘Tully Monster' is a vertebrate. Nature, 532, 496–499. https://doi.org/10.1038/nature16992 26982721 10.1038/nature16992

[ece33941-bib-0031] Morris, S. C. , & Caron, J.‐B. (2014). A primitive fish from the Cambrian of North America. Nature, 512, 419–422. https://doi.org/10.1038/nature13414 24919146 10.1038/nature13414

[ece33941-bib-0032] Nee, S. , & May, R. M. (1997). Extinction and the loss of evolutionary history. Science, 278, 692–694. https://doi.org/10.1126/science.278.5338.692 9381180 10.1126/science.278.5338.692

[ece33941-bib-0033] Nixon, K. C. , & Wheeler, Q. D. (1992). Extinction and the origin of species In Wheeler Q. D., & Novacek M. J. (Eds.), Extinction and Phylogeny (pp. 119–143). New York, NY: Columbia University Press.

[ece33941-bib-0034] O'Leary, M. A. , & Kaufman, S. (2011). MorphoBank: Phylophenomics in the “cloud”. Cladistics, 27, 529–537. https://doi.org/10.1111/j.1096-0031.2011.00355.x 10.1111/j.1096-0031.2011.00355.x 34875801

[ece33941-bib-0035] Paradis, E. , Claude, J. , & Strimmer, K. (2004). APE: Analyses of phylogenetics and evolution in R language. Bioinformatics, 20, 289–290. https://doi.org/10.1093/bioinformatics/btg412 14734327 10.1093/bioinformatics/btg412

[ece33941-bib-0036] Pavoine, S. , Ollier, S. , & Dufour, A. B. (2005). Is the originality of a species measurable? Ecology Letters, 8, 579–586. https://doi.org/10.1111/j.1461-0248.2005.00752.x

[ece33941-bib-0037] Pol, D. , & Escapa, I. H. (2009). Unstable taxa in cladistic analysis: Identification and the assessment of relevant characters. Cladistics, 25, 515–527. https://doi.org/10.1111/j.1096-0031.2009.00258.x 10.1111/j.1096-0031.2009.00258.x 34879625

[ece33941-bib-0038] R Core Team (2016). R: A language and environment for statistical computing. Vienna, Austria: R Foundation for Statistical Computing.

[ece33941-bib-0039] Redding, D. W. , Hartmann, K. , Mimoto, A. , Bokal, D. , DeVos, M. , & Mooers, A. O. (2008). Evolutionarily distinctive species often capture more phylogenetic diversity than expected. Journal of Theoretical Biology, 251, 606–615. https://doi.org/10.1016/j.jtbi.2007.12.006 18313078 10.1016/j.jtbi.2007.12.006

[ece33941-bib-0040] Robinson, D. F. , & Foulds, L. R. (1981). Comparison of phylogenetic trees. Mathematical Biosciences, 53, 131–147. https://doi.org/10.1016/0025-5564(81)90043-2

[ece33941-bib-0041] Ronquist, F. , Teslenko, M. , van der Mark, P. , Ayres, D. L. , Darling, A. , Höhna, S. , … Huelsenbeck, J. P. (2012). MrBayes 3.2: Efficient Bayesian phylogenetic inference and model choice across a large model space. Systematic Biology, 61, 539–542. https://doi.org/10.1093/sysbio/sys029 22357727 10.1093/sysbio/sys029 PMC3329765

[ece33941-bib-0042] Sallan, L. , Giles, S. , Sansom, R. S. , Clarke, J. T. , Johanson, Z. , Sansom, I. J. , & Janvier, P. (2017). The ‘Tully Monster'is not a vertebrate: Characters, convergence and taphonomy in Palaeozoic problematic animals. Palaeontology, 60, 149–157. https://doi.org/10.1111/pala.12282

[ece33941-bib-0043] Sansom, R. S. , Freedman, K. , Gabbott, S. E. , Aldridge, R. J. , & Purnell, M. A. (2010). Taphonomy and affinity of an enigmatic Silurian vertebrate, *Jamoytius kerwoodi* White. Palaeontology, 53, 1393–1409. https://doi.org/10.1111/j.1475-4983.2010.01019.x

[ece33941-bib-0044] Schliep, K. P. (2011). phangorn: Phylogenetic analysis in R. Bioinformatics, 27, 592–593. https://doi.org/10.1093/bioinformatics/btq706 21169378 10.1093/bioinformatics/btq706 PMC3035803

[ece33941-bib-0045] Schmich, F. , Szczurek, E. , Kreibich, S. , Dilling, S. , Andritschke, D. , Casanova, A. , … Emmenlauer, M. (2015). gespeR: A statistical model for deconvoluting off‐target‐confounded RNA interference screens. Genome Biology, 16, 220 https://doi.org/10.1186/s13059-015-0783-1 26445817 10.1186/s13059-015-0783-1 PMC4597449

[ece33941-bib-0046] Siddall, M. E. (1995). Another monophyly index: Revisiting the jackknife. Cladistics, 11, 33–56. https://doi.org/10.1111/j.1096-0031.1995.tb00003.x 10.1111/j.1096-0031.1995.tb00003.x 34920599

[ece33941-bib-0047] Stamatakis, A. (2014). RAxML version 8: A tool for phylogenetic analysis and post‐analysis of large phylogenies. Bioinformatics, 30, 1312–1313. https://doi.org/10.1093/bioinformatics/btu033 24451623 10.1093/bioinformatics/btu033 PMC3998144

[ece33941-bib-0048] Tuffley, C. , & Steel, M. (1997). Links between maximum likelihood and maximum parsimony under a simple model of site substitution. Bulletin of Mathematical Biology, 59, 581–607. https://doi.org/10.1007/BF02459467 9172826 10.1007/BF02459467

[ece33941-bib-0049] Valiente, G. (2009). Combinatorial pattern matching algorithms in computational biology using Perl and R. Boca Raton, FL: CRC Press https://doi.org/10.1201/CHMTHCOMBIO

[ece33941-bib-0050] Van Roy, P. , Daley, A. C. , & Briggs, D. E. (2015). Anomalocaridid trunk limb homology revealed by a giant filter‐feeder with paired flaps. Nature, 522, 77–80. https://doi.org/10.1038/nature14256 25762145 10.1038/nature14256

[ece33941-bib-0051] Warren, D. L. , Geneva, A. J. , & Lanfear, R. (2017). RWTY (R We There Yet): An R package for examining convergence of Bayesian phylogenetic analyses. Molecular Biology and Evolution, 34, 1016–1020. 28087773 10.1093/molbev/msw279

[ece33941-bib-0052] Webber, W. , Moffat, A. , & Zobel, J. (2010). A similarity measure for indefinite rankings. ACM Transactions on Information Systems (TOIS), 28, 20 https://doi.org/10.1145/1852102.1852106

[ece33941-bib-0053] Weitzman, M. L. (1998). The Noah's Ark problem. Econometrica, 66, 1279–1298. https://doi.org/10.2307/2999617

[ece33941-bib-0054] Wickham, H. (2015). stringr: Simple, consistent wrappers for common string operations. Retrieved from https://github.com/tidyverse/stringr.

[ece33941-bib-0055] Zhu, M. , Yu, X. , Ahlberg, P. E. , Choo, B. , Lu, J. , Qiao, T. , … Blom, H. (2013). A Silurian placoderm with osteichthyan‐like marginal jaw bones. Nature, 502, 188–193. https://doi.org/10.1038/nature12617 24067611 10.1038/nature12617

